# Thermal-Oxidation Stability of Soybean Germ Phytosterols in Different Lipid Matrixes

**DOI:** 10.3390/molecules25184079

**Published:** 2020-09-07

**Authors:** Jingnan Chen, Dami Li, Guiyun Tang, Jinfen Zhou, Wei Liu, Yanlan Bi

**Affiliations:** College of Food Science and Technology, Henan University of Technology, Zhengzhou 450001, China; chenjingnan813@126.com (J.C.); li1097086920@163.com (D.L.); ttw661125123@163.com (G.T.); 13607643239@126.com (J.Z.); liuwei307@hotmail.com (W.L.)

**Keywords:** soybean germ phytosterols, phytosterols oxidation products, thermal-oxidation stability, lipids medium

## Abstract

The stability of soybean germ phytosterols (SGPs) in different lipid matrixes, including soybean germ oil, olive oil, and lard, was studied at 120, 150, and 180 °C. Results on the loss rate demonstrated that SGPs were most stable in olive oil, followed by soybean germ oil, and lard in a decreasing order. It is most likely that unsaturated fatty acids could oxidize first, compete with consumption of oxygen, and then spare phytosterols from oxidation. The oxidation products of SGP_S_ in non-oil and oil systems were also quantified. The results demonstrated that at relatively lower temperatures (120 and 150 °C), SGPs’ oxidation products were produced the most in the non-oil system, followed by lard, soybean germ oil, and olive oil. This was consistent with the loss rate pattern of SGPs. At a relatively higher temperature of 180 °C, the formation of SGPs’ oxidation products in soybean germ oil was quantitatively the same as that in lard, implying that the temperature became a dominative factor rather than the content of unsaturated fatty acids of lipid matrixes in the oxidation of SGPs.

## 1. Introduction

Phytosterols (PSs) are a group of natural sterols in plants and their chemical structure is the same as that of cholesterol except they have a different side chain. PS has been demonstrated to be capable of reducing plasma total cholesterol (TC) and low-density lipoprotein cholesterol (LDL-C) and effectively reducing the risk of cardiovascular and cerebrovascular diseases [1,2]. In addition, PS has been reported to be anti-inflammatory and anticarcinogenic [3,4,5]. For these reasons, PS has been commercially added into various foods in recent years, such as oils, sausages, bakery products, spreads, cereals, salad dressings, chocolate, and so on.

Like cholesterol, phytosterols are also susceptible to thermal oxidation during the food process or long-term storage [6]. The mechanism of PS oxidation is believed to be a free radical chain reaction. PS is attacked by reactive oxygen species, such as ozone (O_3_), single oxygen (^1^O_2_), and hydroxyl (OH•) [3]. The double bond on PS is a target of free radical attack followed by hydrogen abstraction on the carbon atom in α-position next to the double bond [7,8]. The oxidation products, hydroxyl, epoxy, keto, and triol derivatives, are the main phytosterol oxidation products (POPs) [9]. Previous studies have demonstrated that POPs are associated with apoptosis [10], atherosclerosis, cytotoxicity, and inflammation [11]. Recent studies have found that the triol derivatives are the most cytotoxic, while 7β-hydroxy and 7-keto derivatives are effective in inducing apoptosis in U937 cells [12,13,14,15].

PS widely exists in vegetable oils, nuts, and plant seeds. The thermal-oxidation stability of phytosterols and formation of POPs in vegetable oils are therefore a health concern. The thermal-oxidation of PS is affected by many factors, including the specific structures of phytosterols, unsaturation degree of fatty acids in vegetable oils, food matrixes, temperatures, and processing times [16,17,18]. Xu et al. investigated the effect of steric acid, oleic acid, linoleic, and α-linolenic acid on the oxidation of cholesterol and *β*-sitosterol, finding that the four fatty acids affected the degradation of cholesterol and *β*-sitosterol in a time-dependent manner and their effect was unlikely related to their degree of unsaturation [19]. Johnsson et al. investigated the POPs contents in olive oil, peanut oil, and maize oil and found that after heating at 180 °C for 0~2 h, POPs in olive oil and peanut oil significantly increased, but this did not happen in peanut oil [20]. 

PS in soybean germ oil is relatively higher compared with other oils (3168 mg/100 g) [21]. No study to date has evaluated the thermal-oxidation stability of soybean germ phytosterols (SGPs) and the formation of their oxidized derivatives. The aims of the present study were (i) to investigate the loss rate of SGPs in a non-oil system and different oil systems (lard, soybean germ oil, and olive oil), and (ii) to characterize the composition of the major oxidation products of SGPs.

## 2. Results and Discussion

### 2.1. Oxidation of SGPs in the Non-Oil System

**Figure 1:**
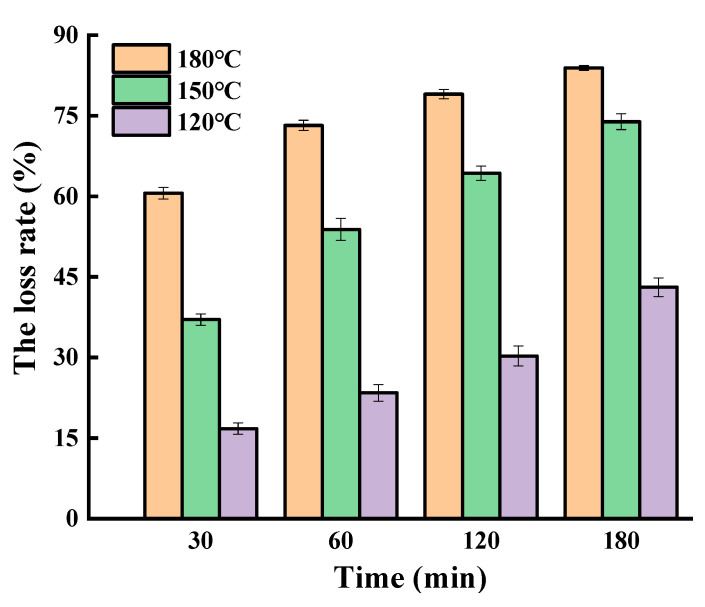
The loss rate of soybean germ phytosterols in the non-oil system.

The loss rate of SGPs in the non-oil system increased with the increasing temperatures and prolonged time (Figure 1). When the heating time increased from 30 to 180 min, the loss rates of SGPs increased from 16.78% to 43.08% at 120 °C, 37.05% to 73.89% at 150 °C, and 60.59% to 83.89% at 180 °C, respectively. The higher the temperatures are, the greater the formation of free radicals. The free radicals produced would attack the double bond on SGPs, resulting in the formation of SGPs’ oxidation products [22]. When SGPs were heated at 180 °C for 30, 60, 120, and 180 min, the loss rates of SGPs were 60.59%, 73.21%, 79.03%, and 83.89%, respectively. This was in agreement with the report of Xu et al., who investigated the oxidative stability of β-sitosterol in a mix with edible oils, finding that, after heating at 180 °C for 120 min, a 75% degradation of β-sitosterol was observed [19]. Wawrzyniak et al. investigated PS degradation in rapeseed stored under different temperatures and humidity. The results indicated that 61% of PS was lost when the seeds (aw = 0.90) were stored at 30 °C for 48 days [23].

### 2.2. Oxidation of SGPs in Oil Systems

**Figure 2:**
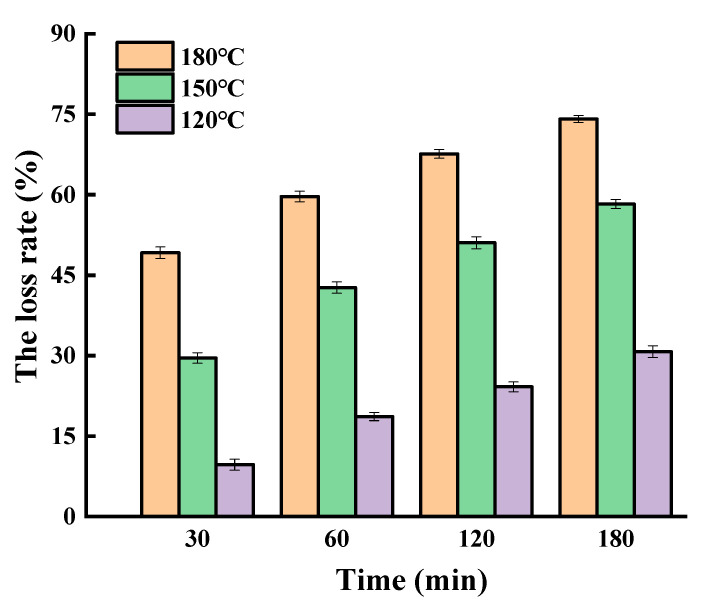
The loss rate of soybean germ phytosterols in soybean germ oil.

The thermal-oxidation stability of SGPs in oils is determined by several factors, including the unsaturation degree of fatty acids, tocopherols, and some other micro-components in oils. The same as in the non-oil system, the loss rate of SGPs in soybean germ oil was time and temperature dependent (Figure 2). To be specific, the loss rates of SGPs at 30, 60, 120, and 180 min at 150 °C were 29.53%, 42.68%, 51.03%, and 58.27%, respectively. When the oils were heated for 30 min, the loss rates of SGPs at 120, 150, and 180 °C reached 9.71%, 29.53%, and 49.18%, respectively. Compared with Figure 1, the loss rate of SGPs in soybean germ oil was lower than that in the non-oil system. When heated at 120, 150, and 180 °C for 180 min, the loss rates of SGPs in soybean germ oil were 30.73%, 58.27%, and 74.12%, respectively, while the corresponding loss rates in the non-oil system increased by 12.35%, 15.62%, and 9.77%, respectively. When heated for 60 min, the loss rates of SGPs were 18.63%, 42.68%, and 59.66%, respectively, while those in the non-oil system increased by 4.81%, 11.19%, and 13.55%, respectively. Barriuso et al. evaluated the effect of different fatty acid methyl esters on the mixture of three plant sterols [24]. The results showed that the presence of fatty acid methyl esters of stearate, oleate, linoletate, and linolenate could delay phytosterols’ degradation and reduced the formation of oxysterols. Together with our results, it could be concluded that the lipid matrix has some protective effect on the degradation of phytosterols.

**Figure 3:**
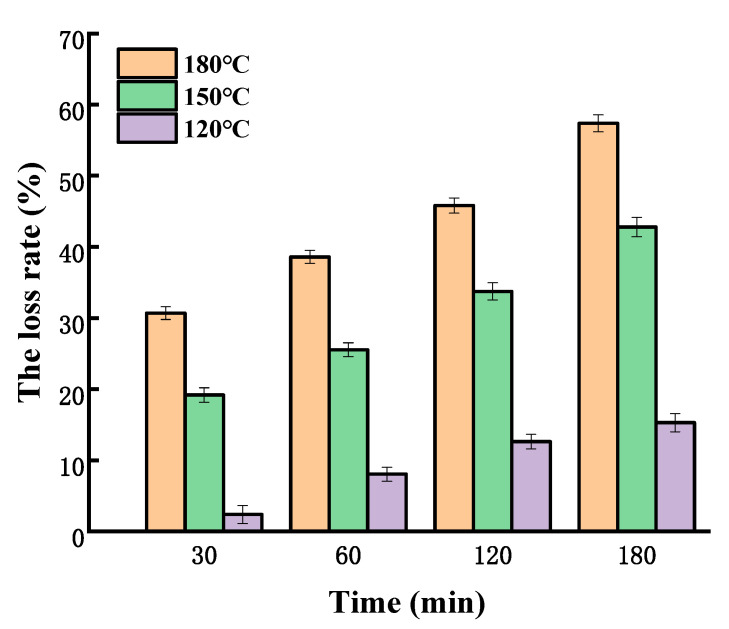
The loss rate of soybean germ phytosterols in olive oil.

**Figure 4:**
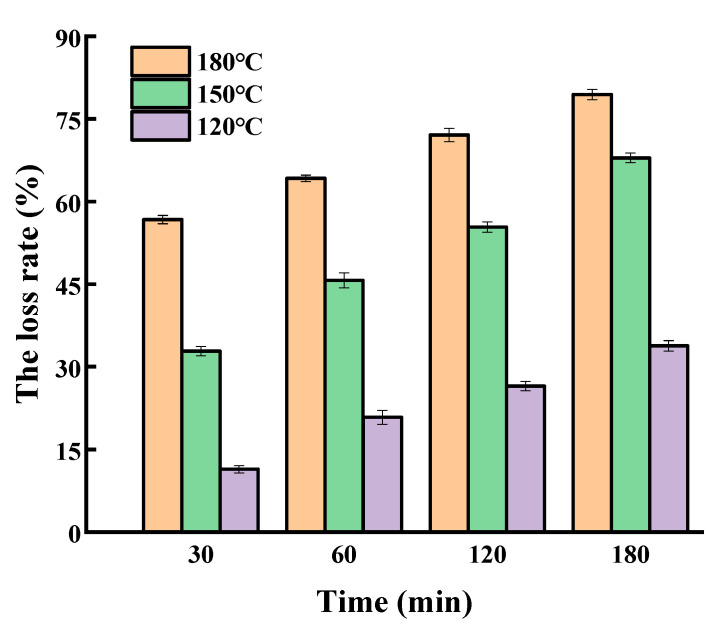
The loss rate of soybean germ phytosterols in lard.

The loss rate of SGPs in olive oil is presented in Figure 3. The results show that the loss rate of the olive oil system was much lower than that in soybean germ oil. After heating for 180 min, the loss rates of SGPs in olive oil at 120, 150, and 180 °C were 15.28%, 45.18%, and 57.37%, respectively, while the corresponding levels in soybean germ oil increased by 14.45%, 13.09%, and 16.75%, respectively, suggesting that SGPs were relatively more stable in olive oil than in soybean germ oil. Lard has up to approximately 40% of saturated fatty acids. The loss rate of SGPs in lard was also investigated (Figure 4). The same as in the soybean germ oil and olive oil system, the loss rate of SGPs in lard was also temperature and time dependent. It was found that the loss rate of SGPs in lard was greater than that in soybean germ oil or olive oil. When heated at 180 °C for 180 min, the loss rates of SGPs in lard, soybean germ oil, and olive oil were 79.44%, 74.12%, and 57.37%, respectively; at 150 °C for 180 min, they were 67.94%, 58.27%, and 42.79%, respectively; and at 120 °C for 180 min, they were 33.79%, 30.73%, and 15.28%, respectively. The loss rates of SGPs in the non-oil system for 180 min at 180, 150, and 120 °C were 83.89%, 73.89%, and 43.08%, respectively. It was concluded that SGPs were most unstable in the non-oil system followed by in lard, soybean germ oil, and olive oil in a decreasing order.

The present study clearly demonstrated that lipid matrixes are an essential factor on the stability of SGPs. In this regard, Kemmo et al. found that the unsaturation degree of the matrix increased the oxidative stability of sterols [25], indicating that unsaturated fatty acids have a certain protective effect on the loss of SGPs. A study conducted by Hu and Chen showed that cholesterol presented higher loss and oxidation products in stearic acid methyl ester than that in linoleic acid methyl ester or docosahexaenoic acid methyl ester [26]. The unsaturated fatty acids in lard, soybean germ oil, and olive oil were 53.91%, 81.53%, and 85.09%, which were inversely correlated with the oxidative stability of SGPs. This suggests that the higher the unsaturation degree of surrounding lipids matrix is, the lesser physterol oxidation that took place [24]. We speculated that unsaturated fatty acids might compete with the oxygen, degrade first, and then spare sterols from oxidation. In addition, temperature had a significant effect on the loss of SGPs both in non-oil and oil systems.

### 2.3. GC-MS Analysis of SGPs Oxidation Products

**Figure 5:**
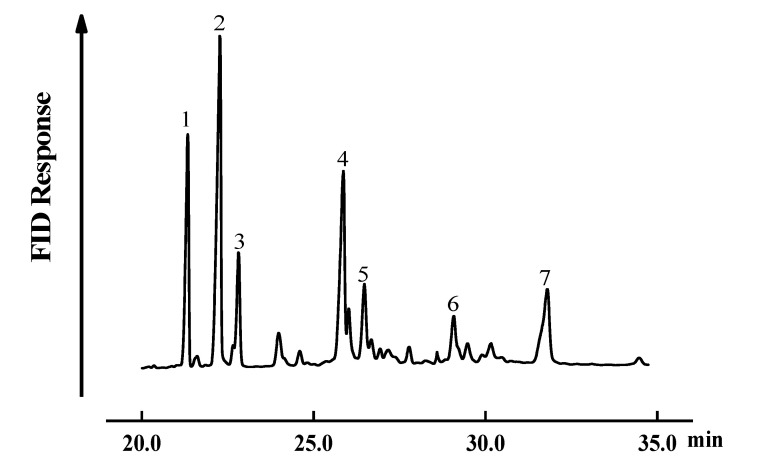
Gas chromatogram analysis of soybean germ phytosterols’ oxidation products (note: 1, cholesterol; 2 and 3, 7 α(β)-hydroxy; 4 and 5, 5 α(β), 6 α(β)-epoxy; 6, triol; 7, 7-keto).

Phytosterols’ oxidation products mainly refer to hydroxides, epoxy sterol oxides, keto oxides, and triol oxides. As shown in Figure 5, peak 1 was the standard 5α-cholestane used for quantification, wherein peaks 2 and 3 represent 7α(β)-hydroxy, 4 and 5 represent 5α(β), 6α(β)-epoxy, 6 represents triol, and 7 represents 7-keto derivatives. The MS fragment chromatogram of SGPs’ oxidation products is shown in upplementary Material (Figure S1), which was mainly classified according to characteristic fragment peaks [25,27], wherein A and B were 7α(β)-hydroxysterol oxides with the characteristic fragment peaks of 482 and 572; C was 7-ketosterol oxides with the characteristic fragment ion peaks of 386, 455, and 498; D and E were 5α(β), 6α(β)-epoxy sterol oxides with the characteristic fragment peaks of 410 and 500; and F was triol oxides with the characteristic fragment ion peaks of 372 and 429.

### 2.4. Composition of SGPs Oxidation Products in the Non-Oil System

Phytosterols and cholesterol undergo a similar process of oxidation and degradation [18]. The thermal oxidation reaction of SGPs is a free radical chain reaction, leading to the formation of 7α(β)-sterol hydroperoxide, then decomposition to 7α(β)-hydroxyl oxide (reduction) and 7-ketosterol oxide (oxidation), and 5α(β), 6α(β)-epoxysterol oxide [28]. SGPs’ oxidation products in the non-oil system at three temperatures of 120, 150, and 180 °C are shown in Table 1. The results show that SGPs’ oxidation products, including 7α(β)-hydroxysterols, 5α(β), 6α(β)-epoxysterols, 7-ketosterols, and triols, increased with the increasing temperature and time. When SGPs were heated from 30 to 180 min, SGPs’ oxidation products increased from 6.87% to 26.05% at 180 °C, 4.48% to 12.73% at 150 °C, and 3.80% to 8.52% at 120 °C, respectively, indicating that the increasing temperatures accelerated the propagation and hydroperoxide decomposition reactions, thus promoting the formation of phytosterols’ oxidation products [22,29]. In addition, 7-ketosterols and 7α(β)-hydroxylsterols were higher, followed by 5α(β), 6α(β)-epoxysterols and triols at 120 and 150 °C. When the temperature was elevated to 180 °C, 5α(β), 6α(β)-epoxysterols and 7-ketosterols were increased significantly. The level of oxidation products was in the order of 7-ketosterols > 5α(β), 6α(β)-epoxysterols > 7α(β)-hydroxylsterols > triols, indicating that 7α(β)-hydroxysterol undergoes further oxidation to form 7-ketosterols and 5α(β), 6α(β)-epoxysterol [30].

### 2.5. The Composition of Soybean Germ Phytosterols’ Oxidation Products in the Oil System

The contents and compositions of SGPs’ oxidation products in different oil systems (soybean germ oil, olive oil, and lard) were also examined (Table 2, Table 3 and Table 4). The results show that the SGPs oxidation products in oils were also composed of 7α(β)-hydroxysterols, 5α(β), 6α(β)-epoxysterols, 7-ketosterols, and triols except for in olive oil, in which triols were absent. The same as in the non-oil system, SGPs’ oxidation products increased with the elevating temperatures and prolonged time. For instance, in soybean germ oil, the total oxidation product at 150 °C was 2.55% (30 min) < 4.59% (60 min) < 6.51% (120 min) < 9.30% (180 min); when the heating time was extended from 30 to 180 min, the total oxidation products increased from 1.55% to 6.27% at 120 °C; 2.55% to 9.30% at 150 °C; 4.94% to 17.10% at 180 °C, respectively. Quantitatively, 7-ketosterols were produced the most followed by 7α(β)-hydroxylsterols, 5α(β), 6α(β)-epoxysterols, and triols (absent in olive oil) except for in soybean germ oil at 180 °C, 5α(β), 6α(β)-epoxysterols were the second most produced followed by 7α(β)-hydroxylsterols. In addition, 7-ketosterols and 5α(β), 6α(β)-epoxysterols increased significantly, while 7α(β)-hydroxylsterol and triol sterol had no further changes. With the temperature increasing from 120 to 180 °C (180 min), the amounts of 7-ketosterols and 5α(β), 6α(β)-epoxysterols in soybean germ oil increased from 3.95% to 10.18% and 0.63% to 4.76%, respectively, while 7α(β)-hydroxylsterols and triols changed from 1.56% to 1.55% and 0.13% to 0.20%, respectively. The same trend was observed in lard. Different changes were seen in olive oil: 7-ketosterols still remained the most produced (from 3.31% to 8.79%) compared with other oxidation products, while 7α(β)-hydroxylsterols and 5α(β), 6α(β)-epoxysterols both had a little increase (from 1.36% to 2.11% and 1.05% to 1.34% respectively). Notably, triols were absent in olive oil. Rudzińska et al. evaluated the oxidative derivatives of phytosterols and phytostanols in margarine at 4 and 20 °C for 18 weeks, finding that 7α(β)-hydroxy was the major oxidation products at lower temperatures [31]. In combination with our results, it is concluded that a lower activation energy is required for hydroxides’ formation [32].

**Figure 6:**
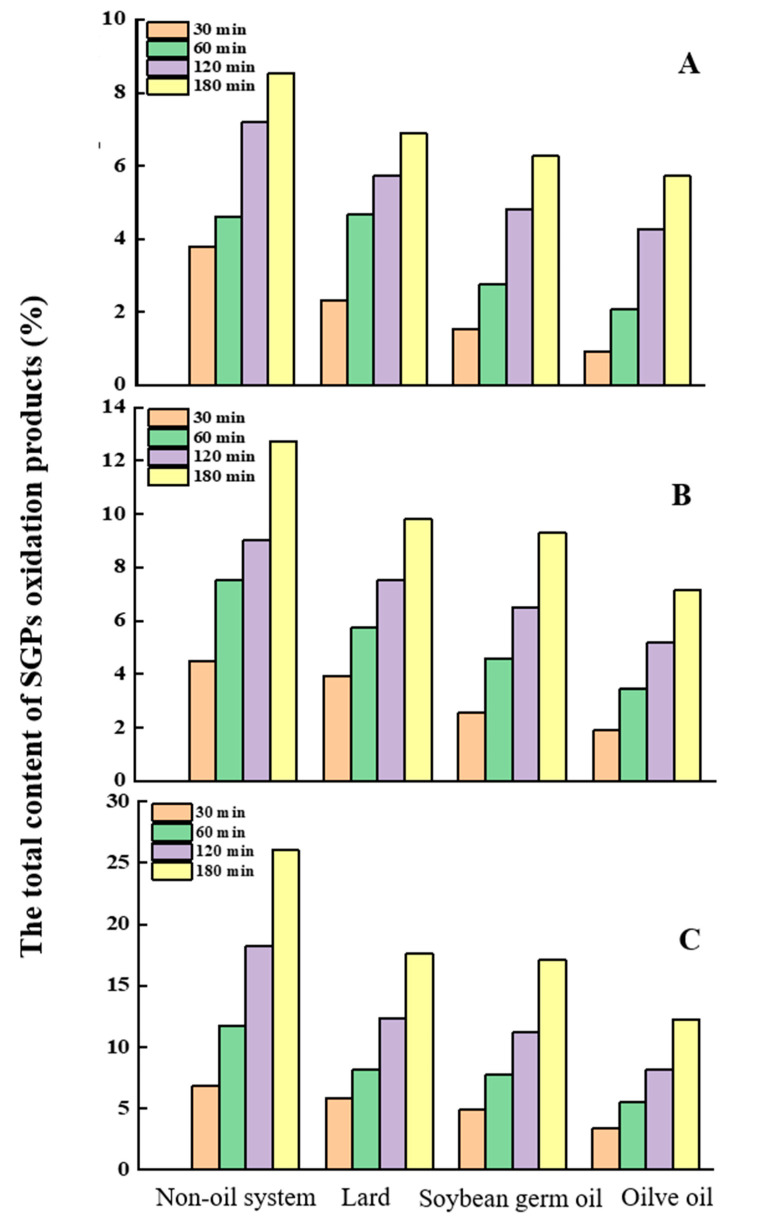
The content of total SGPs’ oxidation products in different systems ((**A**), 120 °C; (**B**), 150 °C; (**C**), 180 °C).

The comparison of the formation of SGPs oxidation products in different systems is shown in Figure 6. The results demonstrated that at relatively lower temperatures (120 and 150 °C) (Figure 6A,B), the total SGPs’ oxidation products were produced in an order of the non-oil system > lard > soybean germ oil > olive oil. For instance, after being heated at 120 °C (30~180 min), the contents of SGPs in the non-oil system, lard, soybean germ oil, and olive oil ranged from 3.80~8.52%, 2.32~6.90%, 1.55~6.27%, and 0.94~5.72%; at 150 °C, the corresponding figures were 4.48~12.73%, 3.94~9.80%, 2.55~9.30%, and 1.89~7.14%. The results indicated that the lipid matrix had an important influence on the formation of sterol oxidation products. Our hypothesis is that the unsaturated fatty acids in the lipid matrix are more readily oxidized at a high temperature, protecting the oxidation of phytosterols to a certain extent [17]. The unsaturated fatty contents of soybean germ oil, olive oil, and lard were 81.53%, 85.09%, and 53.91%, respectively. In relatively saturated lipid matrices (lard), the degree of oxidation of sterols was greater than that in relatively unsaturated lipid systems (soybean germ oil and olive oil). The results are consistent with the study conducted by Ansorena et al. [33]. However, when the temperate was elevated to 180 °C, a different situation was observed (Figure 6C). At the beginning of the heating (180 °C, 30 min), the formation of SGPs’ oxidation products in lard and soybean germ oil was 5.83% and 4.94%, respectively, whereas with the heating time prolonged to 180 min, the difference between the total content of SGPs’ oxidation products in lard and soybean germ oil became less and less (60 min, 7.77% and 8.17%; 120 min, 11.23% and 12.32%; 180 min, 17.10% and 17.64%), implying that the surrounding fatty acids influenced the production of SGPs’ oxidation in a temperature-dependent manner. Pennisi-Forell et al. examined the oxidative stability of low-fat beef burgers formulated with pre-emulsified vegetable and fish oils (10%), tocopherols, and phytosterols, indicating that the concentration of total phytosterols was unaffected by the formulation or storage time at −20 °C [34]. We speculated that at the early stage of oxidation, the unsaturated fatty acids truly had a protective effect on SGPs oxidation, but the thermal-oxidation of the lipid matrix and phytosterols was an interaction process, meaning that the oxidation products of lipids might influence the formation of POPs. Consequently, when the lipid medium was oxidized to a certain degree, the protective effect became weaker, and even disappeared. The major polyunsaturated fatty acid in soybean germ oil was linolenic acid (16.21%), while in lard and olive oil it was linoleic acid, accounting for 12.73% and 4.95%, respectively. The study conducted by Botelho et al. indicated that phytosterol-enriched dark chocolate presented higher values of hydroperoxides, which could be attributed to presence of higher levels of alpha-linolenic acid [35]. Accordingly, soybean germ oil was easily oxidized compared with lard and olive oil. Though the unsaturated fatty acid content of soybean germ oil and olive oil (81.53% and 85.09%) had a minor difference, the formation of SGPs’ oxidation products in soybean germ oil was significantly higher than that in olive oil. On the other hand, the natural antioxidant ingredient (VE) in soybean germ oil was relatively higher (1930 mg/kg) than others and might have a protective role on the oxidation deterioration of sterols [36].

## 3. Materials and Methods

### 3.1. Chemicals and Reagents

Soybean germ was purchased from Riotto Botanical Co. Ltd. (Xi’an, China) and stored at −10 °C before analysis. Lard (no exogenous antioxidants) was obtained from Tianjin Jiuyuan Oil Company (Tianjin, China). Olive oil (first grade, Olivoilà brand) was purchased from a local supermarket. The fatty acid composition, tocopherols, and phytosterols content of soybean germ oil, olive oil, and lard were analyzed. The results were as follows: Soybean germ oil (C_16:0_: 12.59%, C_18:0_: 3.27%, C_18:1_: 9.87%, C_18:2_: 55.45%, C_18:3_: 16.21%; phytosterols, 3081 mg/100g; tocopherol, 1930 mg/kg); olive oil (C_16:0_: 10.33%, C_18:0_: 3.70%, C_18:1_: 79.72%, C_18:2_: 4.95%, C_18:3_: 0.42%; phytosterols, 145.49 mg/100g; tocopherol, 209.95 mg/kg); lard (C_14:0_: 1.50%, C_16:0_: 26.34%, C_18:0_: 17.35%, C_18:1_: 40.28%, C_18:2_: 12.73%, C_18:3_: 0.90%; phytosterols and tocopherol, no detected). Ether, chloroform, acetone, ethanol, and pyridine were purchased from Tianjin Kermel Company (Tianjin, China). 5α-Cholestane, N, O-bis (trimethylsily) trifluoroacetamide (BSTFA), +1% trimethylchlorosilane (TMCS) were all purchased from Sigma-Aldrich Co. (St. Louis, MO, USA). Silica used for TLC plate preparation was obtained from Qingdao Ocean Chemical Factory (Qingdao, China).

### 3.2. Preparation of Soybean Germ Oil and SGPs

Soybean germ was dried in an oven at 60 °C under vacuum, and then was thoroughly ground and sieved using 80 mesh. The soybean germ powder (500 g) was soaked in petroleum ether at a solvent ratio of 10:1 (mL/g) for 24 h. After the mixture was filtered, the afforded cake was soaked again. Then, the solvent was collected and evaporated, and the soybean germ oil was obtained. The resultant soybean germ oil was weighted, and the yield was 11.2%. Soybean germ oil (50 g) was saponified with 1 M potassium hydroxide (500 mL) in ethanol (90 °C, 90 min). Unsaponifiable fraction was extracted with 50 mL n-hexane for three times. After the solvent was removed, crude SGPs was obtained. The crude product was purified on a TLC using a solvent mixture of hexane: ethyl ether: acetic acid (70:30:2, v/v/v). After purification, the purity of SGPs was higher than 95%. The content of phytosterols in soybean germ oil was 3.08%.

### 3.3. Quantification of SGPs

SGPs were firstly converted to their corresponding trimethylsilyl derivatives and then analyzed on gas chromatograph as we previously described [21]. 5α-cholestane was used as internal standard. The intimal column was set at 285 °C for 20 min. The injection and flame ionization detector (FID) temperatures were set at 300 and 360 °C, respectively, with the split flow ratio 20:1. The injection sample volume was 1 μL. Nitrogen was used as carrier gas at the speed of 1.0 mL/min. The sum of the mass of individual phytosterols was the total SGPs mass.

### 3.4. Oxidation of SGPs in a Non-Oil System

SGPs were prepared into 2 mg/mL n-hexane solution, then 2 mL of the solution was accurately transformed into a high-temperature resistance tube and dried under a gentle stream of nitrogen. The tube was heated in an oven at three temperatures of 120, 150, and 180 °C. The sample (1 mg) was taken periodically at 30, 60, 120, and 180 min, respectively. After cooling down, the remaining phytosterols was converted to TMS derivatives with the addition of 0.5 mg 5α-cholestane as an internal standard. The mixture was dried by nitrogen. The resultant SGPs-TMS derivatives were dissolved in 600 uL of hexane for GC analysis.

The thermal loss rate of the SGPs was calculated according to the following Formula (1), where m_1_ represents the mass of the addition amount of SGPs, mg; and m_2_ represents the remaining mass of SGPs after thermal-oxidation, mg:

(1) $$\mathrm{The}\;\mathrm{loss}\;\mathrm{rate}\;\mathrm{of}\;\mathrm{SGPs}=\frac{m1-m2}{m1}\times 100\%\;$$

### 3.5. Oxidation of SGPs in Three Oil Systems

Soybean germ oil (5 g) was heated in an oven at three temperatures (120, 150, and 180 °C). The sample (200 mg) was periodically taken and accurately weighed into a 25-mL flask at 30, 60, 120, and 180 min, respectively. The oil samples were saponified with 1 M potassium hydroxide (2 mL) in ethanol (80 °C, 90 min). After the reaction was completed, 5 mL of distilled water were added. The unsaponificable matter was extracted 3 times with 10 mL of n-hexane. The solvent was combined and then was removed by the rotary evaporator. The resultant SGPs were purified by the TLC method with n-hexane: ether: acetic acid = 70:30:2 (V/V/V) as the developing solvent. Then, the phytosterols were derivatized by TMS with the addition of 0.5 mg 5α-cholestane as internal standard. After being dried by nitrogen, the resultant SGPs-TMS derivatives were dissolved in 600 μL of hexane for GC analysis. The thermal-loss rate of the SGPs in soybean germ oil was calculated according to the same equation used in the non-oil system.

SGPs were added into olive oil and lard, respectively, to adjust the amount of SGPs to reach that in soybean germ oil (3.08%). The oxidation of SGPs in lard and olive oil was similarly investigated using the same experimental procedure and analysis method conducted in soybean germ oil.

### 3.6. Measurement of Soybean Germ Phytosterols’ Oxidation Products

The SGPs’ oxidation products were prepared using the method described by Dutta et al. with slight modification [37]. Pure SGPs (30 mg) were accurately weighed into a high-temperature-resistant tube and were heated in an oven at three temperatures of 120, 150, and 180 °C. The sample (1 mg) was taken at 30, 60, 120, and 180 min, respectively. After cooling to room temperature, the sample was dissolved in 1 mL of n-hexane. The oxidation products were purified by the SiOH solid-phase extraction (SPE) method. First, a mixture solution of ethyl ether and n-hexane (9:1, (V/V)) was used to eliminate the non-polar components, and then the mixture of n-hexane and ethyl ether (1:1, (V/V)) was used to remove the unoxidized SGPs. The oxidized products of SGPs were eluted using 5 mL of acetone. Then, the solvent was dried under nitrogen with 5α-cholestane (2 μg) addition as the internal standard. The resultant SGPs’ oxidation products were converted to TMS (trimethylsilyl) derivatives by N, O-bis (trimethylsilyl) trifluoroacetamide (BSTFA) + 1% trimethylchlorosilane (TMCS) (600 μL, 1 h, 60 °C) for GC-MS analysis.

In the oil systems (soybean germ oil, olive oil, and lard), after heating at a set temperature for a certain time, 1 g of oil was accurately weighed in a 25-mL round-bottom flask. The oil sample was saponified in potassium hydroxide ethanol solution (10 mL, 1 M) at room temperature for 12 h. Then, the unsaponified matter was extracted by n-hexane for 3 times. The solvent was combined and removed by rotating evaporation. Then, the oxidation products were obtained according to the purification method described in the non-oil system.

### 3.7. Analysis of Oxidation Products of SGPs

The analysis of SGPs’ oxidation products was performed by GC-MS as described by Menendez-Carreno et al. with slight modification [38]. The phytosterols’ oxidation products were identified on a GC7890 (Agilent Technologies, Palo Alto, CA, USA) with a chromatographic column HP-5 (30 m × 320 μm × 0.25 μm). Nitrogen was used as a carrier gas (1.0 mL/min) with the split flow ratio of 10:1. The column temperature was set at 325 °C. The oven temperature was programmed at 90 °C for 1 min, increased to 270 °C at a rate of 15 °C/min (13 min), then slowly increased to 300 °C at 1.5 °C min. the injection and FID detector temperature was set at 300 and 300 °C, respectively. The MS conditions were as follows: The ion source at 230 °C, four-stage bar temperature 150 °C, ionization voltage 70 Ev, and the scanning range of 33~650 amu.

The mass concentration of the oxidation products was obtained by using the standard curve method made of 5α-cholestane. The content of SGPs oxidation products was calculated according to the following Formula (2), where m_1_ represents the mass of oxidation products, mg; and m_2_ represents the mass of total SGPs in the sample before oxidation, mg:

(2) $$\mathrm{The}\;\mathrm{content}\;\mathrm{of}\;\mathrm{SGPs}\;\mathrm{oxidation}\;\mathrm{products}\left(\%\right)=\frac{m1}{m2}\times 100\%$$

### 3.8. Statistics

All the experiments were performed in triplicate and the results were expressed as means ± standard deviations. Data significance analysis and mapping were performed using SPSS 16.0 (IBM, Armonk, New York, NY, USA) and Origin 8.5 software, respectively, with *p* < 0.05 indicating significant differences in the results.

## 4. Conclusions

The present study clearly demonstrated that SGPs were most oxidized in a non-oil system followed by in lard, soybean germ oil, and olive oil in a decreasing order, suggesting that lipid matrixes are a crucial factor in the oxidation of phytosterols. Regarding the effect of the fatty acid composition in lipid matrixes on the thermal-oxidation stability of SGPs, the unsaturated fatty acids inhibited the oxidation of SGPs, most likely because they oxidized first, competed with consumption of oxygen, and spared phytosterols from oxidation. At the relatively lower temperatures (120 and 150 °C), SGPs’ oxidation products was produced the most in the non-oil system followed by in lard, soybean germ oil, and olive oil in a decreasing order, accompanied with a similar trend in the loss rate of SGPs. At a relatively higher temperature (180 °C), the formation of SGPs’ oxidation products in soybean germ oil and lard was quantitatively the same, demonstrating that the temperature became a dominative factor rather than the content of unsaturated fatty acids of lipid matrixes in the oxidation of SGPs.

## Figures and Tables

**Table 1:** The total content and composition of SGPs oxidation products in the non-oil system *.

Temperature and Time		The Composition and Content of Oxidation Products/%				
		7α(β)-Hydroxy Sterols	5α(β), 6α(β)- Epoxy Sterols	7-Ketosterols	Triols	Total Content
180 °C	30 min	1.82 ± 0.02 ^a^	1.33 ± 0.02 ^a^	3.61 ± 0.04 ^a^	0.11 ± 0.01 ^a^	6.87 ^a^
	60 min	1.98 ± 0.03 ^b^	3.01 ± 0.03 ^b^	6.62 ± 0.03 ^b^	0.16 ± 0.01 ^ab^	11.77 ^b^
	120 min	2.12 ± 0.02 ^c^	4.92 ± 0.02 ^c^	11.02 ± 0.05 ^c^	0.21 ± 0.02 ^b^	18.27 ^c^
	180 min	2.18 ± 0.04 ^c^	7.42 ± 0.03 ^d^	16.23 ± 0.03 ^d^	0.22 ± 0.02 ^b^	26.05 ^d^
150 °C	30min	1.52 ± 0.03 ^a^	0.52 ± 0.01 ^a^	2.16 ± 0.05 ^a^	0.28 ± 0.01 ^a^	4.48 ^a^
	60 min	1.73 ± 0.06 ^a^	0.82 ± 0.02 ^b^	4.64 ± 0.04 ^b^	0.34 ± 0.01 ^a^	7.53 ^b^
	120 min	1.92 ± 0.03 ^a^	1.30 ± 0.04 ^c^	5.51 ± 0.03 ^c^	0.30 ± 0.02 ^a^	9.03 ^c^
	180 min	2.06 ± 0.41 ^a^	2.65 ± 0.05 ^d^	7.74 ± 0.06 ^d^	0.28 ± 0.03 ^a^	12.73 ^d^
120 °C	30 min	1.28 ± 0.02 ^a^	0.28 ± 0.02 ^a^	1.92 ± 0.03 ^a^	0.32 ± 0.03 ^bc^	3.80 ^a^
	60 min	1.37 ± 0.10 ^a^	0.43 ± 0.02 ^a^	2.63 ± 0.07 ^b^	0.18 ± 0.04 ^a^	4.61 ^b^
	120 min	2.06 ± 0.08 ^b^	0.94 ± 0.06 ^b^	3.99 ± 0.04 ^c^	0.21 ± 0.03 ^ab^	7.20 ^c^
	180 min	2.05 ± 0.02 ^b^	0.91 ± 0.04 ^b^	5.18 ± 0.03 ^d^	0.38 ± 0.03 ^c^	8.52 ^d^

* Data are expressed as the mean ± SD; a, b, c, d Means with different superscript letters differ significantly at *p* < 0.05.

**Table 2:** The total content and composition of SGPs’ oxidation products in soybean germ oil *.

Temperature and Time		Composition and Content of Oxidation Products/%				
		7α(β)-Hydroxy Sterols	5α(β), 6α(β)- Epoxy Sterols	7-Ketosterols	Triols	Total Content
180 °C	30 min	0.84 ± 0.01 ^a^	0.98 ± 0.02 ^a^	3.01 ± 0.03 ^a^	0.11 ± 0.02 ^a^	4.94 ^a^
	60 min	1.31 ± 0.02 ^b^	2.23 ± 0.04 ^b^	4.08 ± 0.02 ^b^	0.15 ± 0.03 ^ab^	7.77 ^b^
	120 min	1.55 ± 0.02 ^c^	3.45 ± 0.01 ^c^	6.11 ± 0.04 ^c^	0.12 ± 0.01 ^ab^	11.23 ^c^
	180 min	1.96 ± 0.03 ^d^	4.76 ± 0.03 ^d^	10.18 ± 0.03 ^d^	0.20 ± 0.02 ^b^	17.10 ^d^
150 °C	30 min	0.67 ± 0.04 ^a^	0.23 ± 0.02 ^a^	1.53 ± 0.03 ^a^	0.12 ± 0.02 ^a^	2.55 ^a^
	60 min	1.03 ± 0.03 ^b^	0.48 ± 0.03 ^b^	2.98 ± 0.06 ^b^	0.10 ± 0.03 ^a^	4.59 ^b^
	120 min	1.42 ± 0.02 ^c^	0.94 ± 0.03 ^c^	4.02 ± 0.05 ^c^	0.13 ± 0.04 ^a^	6.51 ^c^
	180 min	1.74 ± 0.11 ^d^	1.56 ± 0.05 ^d^	5.87 ± 0.03 ^d^	0.13 ± 0.03 ^a^	9.30 ^d^
120 °C	30 min	0.38 ± 0.33 ^a^	0.16 ± 0.03 ^a^	0.88 ± 0.06 ^a^	0.13 ± 0.02 ^a^	1.55 ^a^
	60 min	0.89 ± 0.05 ^ab^	0.32 ± 0.03 ^b^	1.47 ± 0.05 ^b^	0.09 ± 0.05 ^a^	2.77 ^b^
	120 min	1.29 ± 0.06 ^b^	0.57 ± 0.02 ^c^	2.21 ± 0.06 ^c^	0.11 ± 0.04 ^a^	4.81 ^c^
	180 min	1.56 ± 0.03 ^b^	0.63 ± 0.03 ^c^	3.95 ± 0.03 ^d^	0.13 ± 0.05 ^a^	6.27 ^d^

* Data are expressed as the mean ± SD; a, b, c, d Means with different superscript letters differ significantly at *p* < 0.05.

**Table 3:** The total content and composition of SGPs’ oxidation products in olive oil *.

Temperature and Time		Composition and Content of Oxidation Products/%				
		7α(β)-Hydroxy Sterols	5α(β), 6α(β)- Epoxy Sterols	7-Ketosterols	Triols	Total Content
180 °C	30 min	0.98 ± 0.07 ^a^	0.58 ± 0.06 ^a^	1.83 ± 0.05 ^a^	ND	3.39 ^a^
	60 min	1.35 ± 0.09 ^a^	0.84 ± 0.05 ^a^	3.29 ± 0.04 ^b^	ND	5.48 ^b^
	120 min	2.03 ± 0.12 ^b^	1.09 ± 0.96 ^a^	5.08 ± 0.11 ^c^	ND	8.20 ^c^
	180 min	2.11 ± 0.11 ^b^	1.34 ± 0.05 ^a^	8.79 ± 0.16 ^d^	ND	12.24 ^d^
150 °C	30 min	0.48 ± 0.08 ^a^	0.20 ± 0.03 ^a^	1.21 ± 0.04 ^a^	ND	1.89 ^a^
	60 min	0.72 ± 0.05 ^b^	0.57 ± 0.07 ^b^	2.15 ± 0.04 ^b^	ND	3.44 ^b^
	120 min	1.11 ± 0.03 ^c^	0.99 ± 0.03 ^c^	3.09 ± 0.06 ^c^	ND	5.19 ^c^
	180 min	1.49 ± 0.05 ^d^	1.28 ± 0.06 ^d^	4.37 ± 0.05 ^d^	ND	7.14 ^d^
120 °C	30 min	0.28 ± 0.04 ^a^	0.13 ± 0.02 ^a^	0.53 ± 0.07 ^a^	ND	0.94 ^a^
	60 min	0.69 ± 0.05 ^b^	0.37 ± 0.01 ^b^	1.03 ± 0.02 ^b^	ND	2.09 ^b^
	120 min	0.94 ± 0.07 ^b^	0.86 ± 0.04 ^c^	2.47 ± 0.05 ^c^	ND	4.27 ^c^
	180 min	1.36 ± 0.11 ^c^	1.05 ± 0.03 ^d^	3.31 ± 0.01 ^d^	ND	5.72 ^d^

* Data are expressed as the mean ± SD; a, b, c, d Means with different superscript letters differ significantly at *p* < 0.05. ND means not detectable.

**Table 4:** The total content and composition of SGPs’ oxidation products in lard *.

Temperature and Time		Composition and Content of Oxidation Products/%				
		7α(β)-Hydroxy Sterols	5α(β), 6α(β)- Epoxy sterols	7-ketosterols	Triols	Total Content
180 °C	30 min	1.03 ± 0.09 ^a^	1.06 ± 0.14 ^a^	3.37 ± 0.13 ^a^	0.37 ± 0.04 ^a^	5.83 ^a^
	60 min	1.49 ± 0.10 ^b^	1.23 ± 0.12 ^ab^	5.13 ± 0.10 ^b^	0.32 ± 0.07 ^a^	8.17 ^b^
	120 min	1.88 ± 0.08 ^bc^	1.78 ± 0.09 ^bc^	8.38 ± 0.09 ^c^	0.28 ± 0.03 ^a^	12.32 ^c^
	180 min	2.12 ± 0.16 ^c^	2.05 ± 0.23 ^c^	13.12 ± 0.17 ^d^	0.35 ± 0.04 ^a^	17.64 ^d^
150 °C	30 min	1.01 ± 0.11 ^a^	0.38 ± 0.12 ^a^	2.28 ± 0.02 ^a^	0.27 ± 0.02 ^a^	3.94 ^a^
	60 min	1.44 ± 0.09 ^ab^	0.57 ± 0.07 ^a^	3.47 ± 0.06 ^b^	0.25 ± 0.03 ^a^	5.73 ^b^
	120 min	1.87 ± 0.18 ^b^	1.27 ± 0.15 ^b^	4.11 ± 0.05 ^c^	0.28 ± 0.05 ^a^	7.53 ^c^
	180 min	2.03 ± 0.22 ^b^	1.68 ± 0.18 ^b^	5.87 ± 0.03 ^d^	0.22 ± 0.08 ^a^	9.80 ^d^
120 °C	30 min	0.62 ± 0.21 ^a^	0.23 ± 0.04 ^a^	1.23 ± 0.16 ^a^	0.24 ± 0.12 ^a^	2.32 ^a^
	60 min	1.03 ± 0.15 ^ab^	0.41 ± 0.13 ^ab^	2.94 ± 0.17 ^b^	0.29 ± 0.03 ^a^	4.67 ^b^
	120 min	1.59 ± 0.07 ^bc^	0.68 ± 0.09 ^bc^	3.23 ± 0.09 ^b^	0.22 ± 0.02 ^a^	5.72 ^c^
	180 min	1.84 ± 0.12 ^c^	0.82 ± 0.11 ^c^	4.06 ± 0.21 ^c^	0.18 ± 0.06 ^a^	6.90 ^d^

* Data are expressed as the mean ± SD; a, b, c, d Means with different superscript letters differ significantly at *p* < 0.05.

## Supplementary Materials

The followings are available online. Figure S1: GC-MS ion fragment chromatogram of soybean germ sterols oxidation products, Table S1: The loss rate of soybean germ phytosterols in different systems, Table S2: The fatty acid compositions, tocopherols and phytosterols contents in different oils.

## References

[1] Jogchum P., Ronald P., Mensink (2005). Plant stanol and sterol esters in the control of blood cholesterol levels: Mechanism and safety aspects. Am. J. Cardiol..

[2] Fernandes P., Cabral J.M.S. (2007). Phytosterols: Applications and recovery methods. Bioresour. Technol..

[3] García-Llatas G., Rodriguez-Estrada M.T. (2011). Current and new insights on phytosterol oxides in plant sterol-enriched food. Chem. Phys. Lipids.

[4] Marangoni F., Poli A. (2010). Phytosterols and cardiovascular health. Pharm. Res..

[5] Alemany C.L., González L.M., García-Llatas G., Alegría A., Barberá R., Sánchez S.L.M., Lagarda M.J. (2012). Sterol stability in functional fruit beverages enriched with different plant sterol sources. Food Res. Int..

[6] Dutta P.C., Dutta P.C. (2004). Chemistry, analysis, and occurrence of phytosterol oxidation products in foods. Phytosterols as Functional Food Components and Nutraceuticals.

[7] Choe E., Min D.B. (2009). Mechanisms of antioxidants in the oxidation of foods. Compr. Rev. Food Sci. Food Saf..

[8] Lengyel J., Rimarčík J., Vagánek A., Fedor J., Lukeš V., Klein E. (2012). Oxidation of sterols: Energetics of C-H and O-H bond cleavage. Food Chem..

[9] O’Callaghan Y., McCarthy F.O., O’Brien N.M. (2014). Recent advances in phytosterol oxidation products. Biochem. Biophys. Res. Commun..

[10] Otaegui-Arrazola A., Menéndez-Carreño M., Ansorena D., Astiasarán I. (2010). Oxysterols: A world to explore. Food Chem. Toxicol..

[11] Alemany L., Barbera R., Alegría A., Laparra J.M. (2014). Plant sterols from foods in inflammation and risk of cardiovascular disease: A real threat?. Food Chem. Toxicol..

[12] Kenny O., O’Callaghan Y., O’Connell N.M., McCarthy F.O., Maguire A.R., O’Brien N.M. (2012). Oxidized derivatives of dihydrobrassicasterol: Cytotoxic and apoptotic potential in U937 and HepG2 cells. J. Agric. Food Chem..

[13] O’Callaghan Y., Kenny O., O’Connell N.M., Maguire A.R., McCarthy F.O., O’Brien N.M. (2013). Synthesis and assessment of the relative toxicity of the oxidized derivatives of campesterol and dihydrobrassicasterol in U937 and HepG2 cells. Biochimie.

[14] O’Callaghan Y.C., Foley D.A., O’Connell N.M., McCarthy F.O., Maguire A.R., O’Brien N.M. (2010). Cytotoxic and apoptotic effects of the oxidized derivatives of stigmasterol in the U937 human monocytic cell line. J. Agric. Food Chem..

[15] Ryan E., Chopra J., McCarthy F.O., Maguire A.R., O’Brien N.M. (2005). Qualitative and quantitative comparison of the cytotoxic and apoptotic potential of phytosterol oxidation products with their corresponding cholesterol oxidation products. Br. J. Nutr..

[16] Smith L.L. (1981). Cholesterol Autoxidation.

[17] Soupas L., Juntunen L., Lampi A.M., Piironen V. (2004). Effects of sterol structure, temperature, and lipid medium on phytosterol oxidation. J. Agric. Food Chem..

[18] Rudzinska M., Przybylski R., Wasowicz E. (2009). Products formed during thermo-oxidative degradation of phytosterols. J. Am. Oil Chem. Soc..

[19] Xu G.H., Sun J.L., Liang Y.T., Yang Y., Chen Z.Y. (2011). Interaction of fatty acids with oxidation of cholesterol and β-sitosterol. Food Chem..

[20] Johnsson L., Dutta P.C. (2006). Determination of phytosterol oxides in some food products by using an optimized transesterification method. Food Chem..

[21] Chen J.N., Tang G.Y., Zhou J.F., Liu W., Bi Y.L. (2019). The characterization of soybean germ oil and the antioxidative activity of its phytosterols. RSC Adv..

[22] Smith L.L. (1996). Review of progress in sterol oxidations: 1987–1995. Lipids.

[23] Wawrzyniak J., Gawrysiak-Witulska M., Rudzińska M. (2019). Dynamics of phytosterol degradation in a bulk of rapeseed stored under different temperature and humidity conditions. J. Stored Prod. Res..

[24] Barriuso B., Astiasarán I., Ansorena D. (2016). Unsaturated lipid matrices protect plant sterols from degradation during heating treatment. Food Chem..

[25] Kemmo S., Ollilainen V., Lampi A. (2007). Determination of stigmasterol and cholesterol oxides using atmospheric pressure chemical ionization liquid chromatography-Mass spectrometry. Food Chem..

[26] Hu P.C., Chen B.H. (2002). Effects of riboflavin and fatty acid methyl esters on cholesterol oxidation during illumination. J. Agric. Food Chem..

[27] Menendez-Carreno M., Ansoreena D., Astiasaran I. (2008). Stability of sterols in phytosterol-enriched milk under different heating conditions. J. Agric. Food Chem..

[28] Iuliano L. (2011). Pathways of cholesterol oxidation via non-enzymatic mechanisms. Chem. Phys. Lipids.

[29] Lundberg W.O., Schultz H.W., Day E.A., Sinnhuber R.O. (1962). Mechanisms in Lipids and Their Oxidation.

[30] Porter N.A., Caldwell S.E., Mills K.A. (1995). Mechanisms of free radical oxidation of unsaturated lipids. Lipids.

[31] Rudzińska M., Przybylski R., Wasowicz E. (2014). Degradation of phytosterols during storage of enriched margarines. Food Chem..

[32] Chien J.T., Hsu D.J., Chen B.H. (2006). Kinetic model for studying the effect of quercetin on cholesterol oxidation during heating. J. Agric. Food Chem..

[33] Ansorena D., Barriuso B., Cardenia V. (2013). Thermo-oxidation of cholesterol: Effect of the unsaturation degree of the lipid matrix. Food Chem..

[34] Pennisi-Forell S.C.P., Ranalli N., Zaritzky N.E., Andres S.C., Califano A.N. (2010). Effect of type of emulsifiers and antioxidants on oxidative stability, colour and fatty acid profile of low-fat beef burgers enriched with unsaturated fatty acids and phytosterols. Meat Sci..

[35] Botelho P.B., Galasso M., Dias V., Mandrioli M., Lobato L.P., Rodriguez-Estrada M.T., Castro I.A. (2014). Oxidative stability of functional phytosterol-enriched dark chocolate. LWT Food Sci. Technol..

[36] Tabee E., Azadmard-Damirchi S., Jägerstad M., Dutta P.C. (2008). Effects of a-tocopherol on oxidative stability and phytosterol oxidation during heating in some regular and high-oleic vegetable oils. J. Am. Oil Chem. Soc..

[37] Dutta P.C., Helmersson S., Kebedu E. (1994). Variation in lipid composition of niger seed (*Guizotia abyssinica*, Cass.) samples collected from different regions in ethiopia. J. Am. Oil Chem. Soc..

[38] Menendez-Carreno M., Garcia-Herreros C., Astiasaran I., Ansorena D. (2008). Validation of a gas chromatography-mass spectrometry method for the analysis of sterol oxidation products in serum. J. Chromatogr. B Anal. Technol. Biomed. Life Sci..

