# Supplementary material for: Thermal-Oxidation Stability of Soybean Germ Phytosterols in Different Lipid Matrixes
Source: Molecules. 2020 Sep 7;25(18):4079. doi: 10.3390/molecules25184079 (PMC7570545; doi:10.3390/molecules25184079)
Supplement: Supplementary file 1 [file molecules-25-04079-s001.pdf]

Supplementary data

Thermal-Oxidation Stability of Soybean Germ Phytosterols in Different Lipid Matrixes

Jingnan Chen[1], Dami Li[1], Guiyun Tang[1], Jinfen Zhou[1], Wei Liu[1], Yanlan Bi[1]*

([1]College of Food Science and Technology, Henan University of Technology, Zhengzhou, 450001, China)

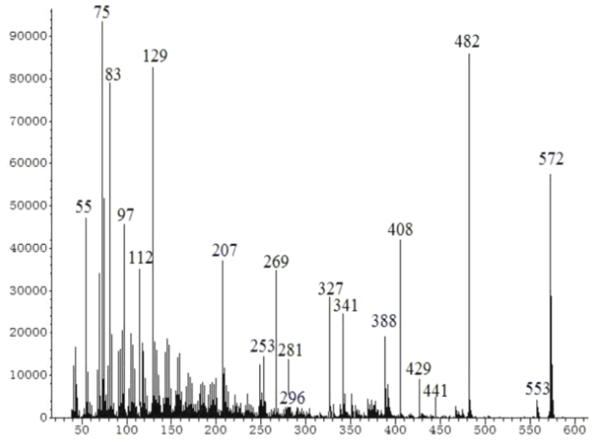

A

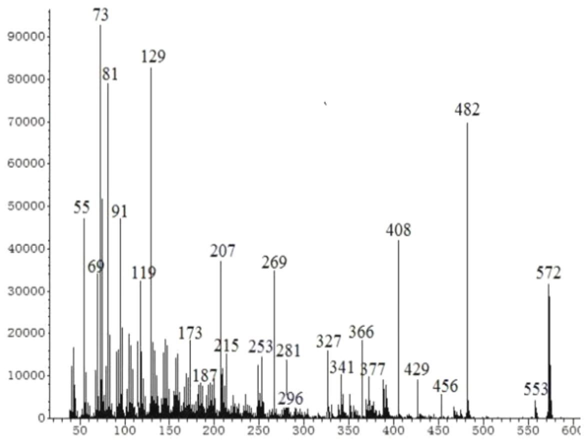

B

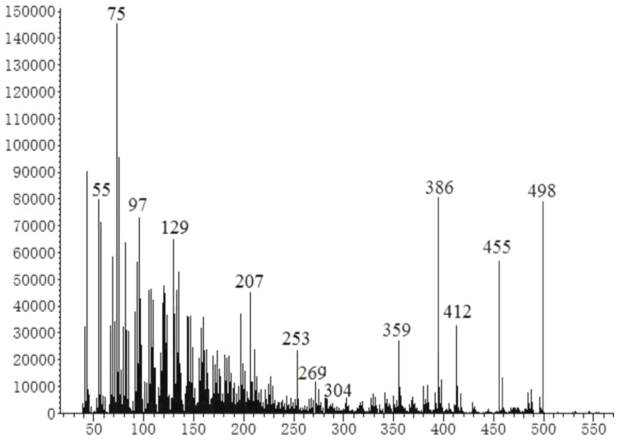

C

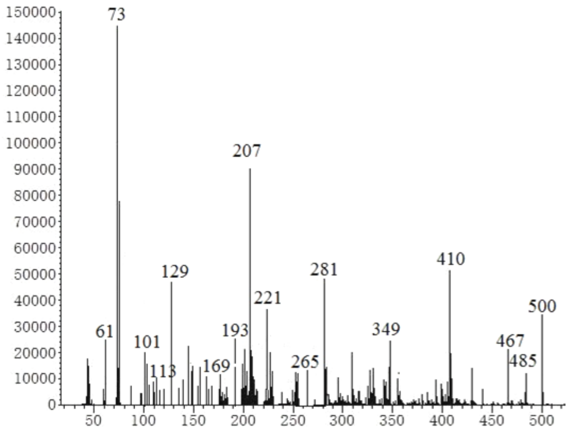

D

[1]The corresponding author: Yanlan Bi at bylzry@l26.com. Tel/Fax: 086-371-67758022

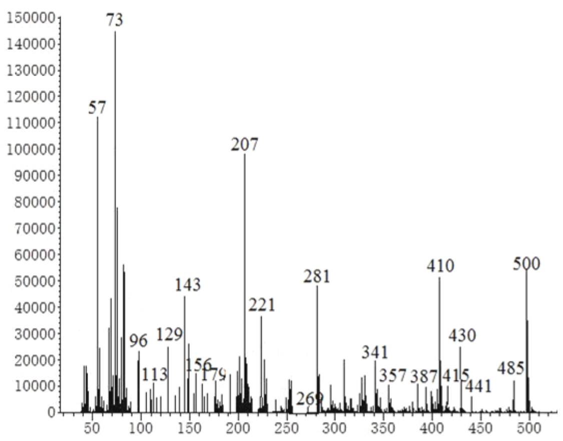

E

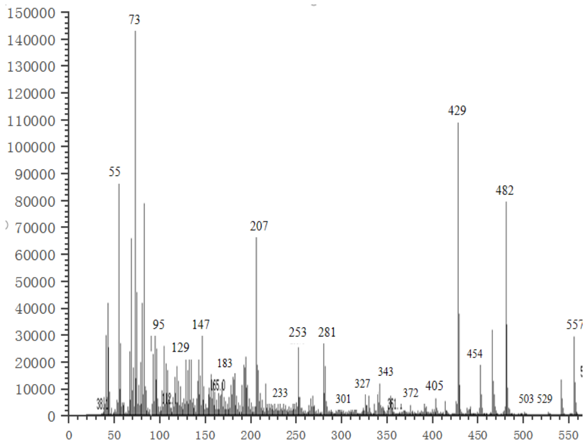

F

Fig. S1 GC-MS ion fragment chromatogram of soybean germ sterols oxidation products

Note: A, 7α-hydroxyl oxides; B, 7β-hydroxyl oxides; C, 7-ketone oxides; D, 5α, 6α-epoxy oxides; E, 5β, 6β-epoxy oxides; F, triols oxides

Table S1 The loss rate of soybean germ phytosterols in different systems

**Temperature and time**		The loss rate of soybean germ phytosterols in different systems/%			
		Non-oil system	Soybean germ oil	Olive oil	Lard
180 °C	30 min	60.59±1.05^{d}	49.18±1.08^{b}	30.68±0.89^{a}	56.73±0.77^{c}
	60 min	73.21±0.94^{d}	59.66±0.99^{b}	38.59±0.92^{a}	64.21±0.59^{c}
	120 min	79.03±0.86^{d}	67.62±0.81^{b}	45.81±1.07^{a}	72.08±1.21^{c}
	180 min	83.89±0.43^{d}	74.12±0.64^{b}	57.37±1.18^{a}	79.44±0.94^{c}
150 °C	30 min	37.05±1.05^{d}	29.53±0.98^{b}	19.18±1.02^{a}	32.84±0.83^{c}
	60 min	53.87±2.04^{c}	42.68±1.06^{b}	25.54±0.97^{a}	45.69±1.34^{b}
	120 min	64.32±1.32^{d}	51.03±1.11^{b}	33.73±1.21^{a}	55.37±0.94^{c}
	180 min	73.89±1.48^{d}	58.27±0.83^{b}	42.79±1.38^{a}	67.94±0.87^{c}
120 °C	30 min	16.78±1.05^{c}	9.71±1.04^{b}	2.38±1.27^{a}	11.39±0.65^{b}
	60 min	23.44±1.54^{c}	18.63±0.76^{b}	8.06±0.99^{a}	20.82±1.27^{c}
	120 min	30.3±1.85^{c}	24.18±0.92^{b}	12.63±1.03^{a}	26.48±0.86^{b}
	180 min	43.08±1.73^{c}	30.73±1.11^{b}	15.28±1.26^{a}	33.79±0.96^{b}

Table S2 The fatty acid compositions, tocopherols and phytosterols contents in different oils

Fatty acid (%)	Soybean germ oil	Olive oil	Lard
Lauric acid (14:0)	ND	ND	1.50
Palmitic acid (16:0)	12.59	10.33	26.34
Stearic acid (18:0)	3.27	3.70	17.35
Oleic acid (18:1)	9.87	79.72	40.28
Linoleic acid (18:2)	55.45	4.95	12.73

Linolenic acid (18:3)	16.21	0.42	0.90
Other acid	0.75	0.88	0.90
Tocopherol/(mg/kg)	1930	209.95	ND
Phytosterol/(mg/100g)	3081	145.49	ND
